# Assessment of Various Process Parameters for Optimized Sterilization Conditions Using a Multi-Sensing Platform

**DOI:** 10.3390/foods11050660

**Published:** 2022-02-24

**Authors:** Farnoosh Vahidpour, Eric Guthmann, Julio Arreola, Yousef Alghazali, Torsten Wagner, Michael J. Schöning

**Affiliations:** 1 Institute of Nano- und Biotechnologies (INB), FH Aachen, 52428 Jülich, Germany; vahidpour@fh-aachen.de (F.V.); eric.guthmann@alumni.fh-aachen.de (E.G.); arreola@fh-aachen.de (J.A.); yousef.alghazali@alumni.fh-aachen.de (Y.A.); torsten.wagner@fh-aachen.de (T.W.); 2 Institute of Biological Information Processing (IBI-3), Research Center Jülich GmbH, 52428 Jülich, Germany

**Keywords:** gaseous hydrogen peroxide, multi-sensing platform, aseptic parameters, sterility, spore kill rate

## Abstract

In this study, an online multi-sensing platform was engineered to simultaneously evaluate various process parameters of food package sterilization using gaseous hydrogen peroxide (H_2_O_2_). The platform enabled the validation of critical aseptic parameters. In parallel, one series of microbiological count reduction tests was performed using highly resistant spores of *B. atrophaeus* DSM 675 to act as the reference method for sterility validation. By means of the multi-sensing platform together with microbiological tests, we examined sterilization process parameters to define the most effective conditions with regards to the highest spore kill rate necessary for aseptic packaging. As these parameters are mutually associated, a correlation between different factors was elaborated. The resulting correlation indicated the need for specific conditions regarding the applied H_2_O_2_ gas temperature, the gas flow and concentration, the relative humidity and the exposure time. Finally, the novel multi-sensing platform together with the mobile electronic readout setup allowed for the online and on-site monitoring of the sterilization process, selecting the best conditions for sterility and, at the same time, reducing the use of the time-consuming and costly microbiological tests that are currently used in the food package industry.

## 1. Introduction

Package sterilization is one of the most important stages of aseptic filling procedures [1,2,3,4], especially in the food industry. The aim is to deliver food products that are safe for the customer and have long-term stability [2,3,5]. To achieve this, several methods have been developed to control the quality of food products, such as milk [6,7,8,9,10,11,12]. However, there is only a little research regarding online and on-site monitoring for controlling the efficiency of package sterilization.

For package sterilization, H_2_O_2_ has become favorable over the past decade as a sterilant for the food and pharmaceutical industries [4,11,13,14] because it decomposes to water and oxygen, which are totally environmentally friendly end products. H_2_O_2_ is either applied in liquid form at lower temperatures or in gas form at elevated temperatures [3,5,15]. The application of H_2_O_2_ is combined with hot air flow or radiation [16,17,18]. When applying H_2_O_2_ in the gas form to sterilize packages, many physical and chemical factors come into play [5,19], including the concentration and temperature of the gas, flow direction, the relative humidity in the sterilization chamber and finally, the efficacy of the sterilant gas on the spore kill rate. These factors influence the quality of the sterilization efficiency. Therefore, setups that control the quality are designed to predefine and check the conditions inside the sterilization chamber [5,20]. Additionally, numerical methods have been applied to analyze and optimize the process parameters during or after sterilization to define the maximal content of residual components, etc. [15,20,21,22,23]. Nevertheless, above all of these control setups and methods, microbiological experiments are also required as a reference method and industrial standard to confirm the sterilization [24,25,26]. Typically, traditional microbiological tests utilize microorganisms, such as *Bacillus atrophaeus* spores, as highly resistant bio-indicators [4]. These tests are frequently used in the industry to guarantee the efficacy of the sterilization process [4,25]. Microbiological tests, such as endpoint testing and count reduction testing [26], can deliver spore viability results in a minimum of 48–72 h [25]. From an industrial point of view, this yields a financial loss at the production and maintenance levels, as the sterile product must be stored until the test results have come back clear. In the case of a negative test result, large amounts of products need to be destroyed because it is impossible to distinguish at which point the sterilization was compromised between the tests. Therefore, looking into other methods to avoid this loss is beneficial in order to find a path toward online and on-site sterilization validation.

Over recent years, more specific methods and systems have been developed to determine optimum sterilization conditions using gaseous H_2_O_2_, including electrochemical, conductometric or colorimetric methods [14,27,28,29]. However, most of these methods cannot be involved in the online or on-site monitoring of sterilization because of transportability, sample preparation, response time or selectivity issues [27,28]. The aim of recent research over the last decade, therefore, has been to convert sterilization assessment into a less costly evaluation, upgrade it to a higher level of online monitoring and, in parallel, speed up the microbiological processes in a way that maintains the consistency of the output [19,30,31].An exemplary piece of research from the same group in 2013 evaluated the individual critical parameters (such as H_2_O_2_ concentration and the exposure time of the spores) in aseptic processing and assessed their relationship with the logarithmic kill rate of spores [32]. This was an important step toward the online monitoring of the sterilization process; however, these parameters have not yet been monitored simultaneously. Another motivating study in 2019 suggested the combination of a calorimetric gas sensor and a spore-based biosensor for sterility validation, which also took into account several critical factors, such as the temperature and concentration of the H_2_O_2_ gas, spore viability and exposure time [19,33]. These attempts have been carried out to validate which conditions have the best effect on the inactivation of the spores (in that case, *B. atrophaeus*). Here, spores were exposed to different H_2_O_2_ concentrations for short periods (~ 2 s) under several gas temperatures and gas flows and then their viability was examined [19,33,34].

The aim of the current study was to implement the simultaneous online control of multiple process parameters that directly influence sterilization. The on-site monitoring of the critical factors in sterilization processes would allow us to determine valid sterilization straightaway. In this way, the industrial losses due to the delays from the time-consuming microbiological tests could be avoided. To follow on from the above-mentioned pioneering research, in the case of package sterilization using gaseous hydrogen peroxide, two additional parameters needed to be considered besides H_2_O_2_ concentration and gas temperature: relative humidity and gas flow direction. These factors, together with exposure time of the spore to the gaseous H_2_O_2_, could influence the sterility efficacy depending on the type of process. Therefore, developing a multi-sensing platform for the assessment of multiple factors was deemed to be useful for the simultaneous online monitoring of those factors. To achieve this, different sensor setups were implemented on a single board to validate the conditions that led to the highest spore kill rate. A flexible calorimetric gas sensor was utilized to detect the temperature and concentration of the gaseous H_2_O_2_ [31], a humidity sensor was applied to record the relative humidity in the sterilization chamber and an array of temperature sensors monitored the flow direction. Additionally, various H_2_O_2_ concentrations and exposure times of the spores to the gaseous H_2_O_2_ were considered. In parallel with the multi-sensing platform, one series of microbiological experiments (by means of count reduction testing) was performed, under the same conditions that were set for the characterization of the multi-sensing platform, in order to validate spore viability. Once the highest spore kill rate has been confirmed by the multi-sensing platform, no more time-consuming traditional microbiological tests would be required. The novelty of this study is, therefore, the application of a single multi-sensing platform to take control of aseptic processing and confirm sterilization validity both online and on-site.

## 2. Materials and Methods

**Figure 1:**
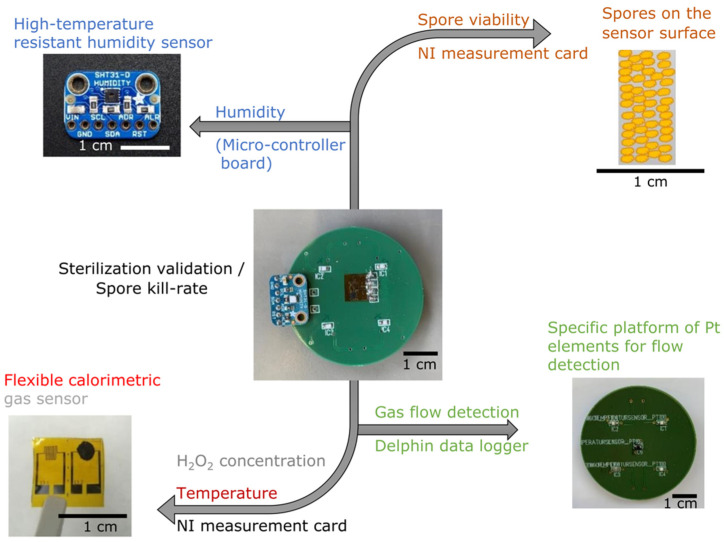
The critical process parameters in sterilization validation that were monitored by means of the developed multi-sensing platform.

The developed multi-sensing platform consisted of multiple electrodes and electrode setups on a printed circuit board (PCB), which could simultaneously detect and record different process parameters in the sterilization process (Figure 1). These electrodes and setups included (i) a flexible calorimetric gas sensor for the detection of H_2_O_2_ temperature and concentration, (ii) a high-temperature resistant humidity sensor for assessing the humidity in the chamber and (iii) an array of Pt100 temperature elements for monitoring the gas flow direction. The arrangement of these sensors on the PCB allowed for an equal and symmetric gas inlet to all sensors. The PCB was mounted inside the chamber of an experimental test rig, where sterilization using H_2_O_2_ gas occurred. Detailed information on the experimental test rig and the positioning of the PCB is provided elsewhere [35,36]. In the sterilization chamber, 35% *w/w* gaseous H_2_O_2_ was applied in the different concentrations of 0, 2.2, 4.1, 5.7, 7.1 and 7.7% *v/v* and 0, 2.2, 4.1, 5.7 and 6.6% *v/v* for the two gas flow rates of 8 and 12 m^3^/h, respectively. Additionally, three initial gas temperatures (210, 240 and 270 °C) were utilized to define various scenarios for the sterilization process (see Table 1 as a reference to the various scenarios).

The readout and analysis of the data from the multi-sensing platform was performed via an Arduino micro-controller, National Instruments (NI) data acquisition (DAQ) cards and a data logger (Delphin). In parallel with these experiments, microbiological tests (count reduction testing) were performed, as a reference method, under the same scenarios as for the operating cycles of the multi-sensing platform in order to validate the spore sterilization and fulfill ongoing industrial standards.

**Figure 2:**
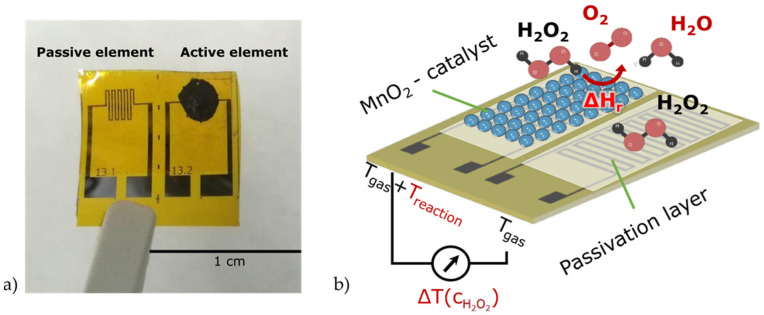
(**a**) A photo of the flexible calorimetric gas sensor with the passive and activated (with MnO_2_) temperature elements. (**b**) The schematically presented exothermic reaction of the H_2_O_2_ with MnO_2_ on the active sensor surface.

A flexible calorimetric gas sensor based on a polyimide sheet was the first sensor implemented in the PCB multi-sensing platform and was used to determine the temperature and concentration of the H_2_O_2_ during the aseptic process (bottom left of Figure 1 and Figure 2a). As reported previously [31], the calorimetric gas sensor was designed as a differential setup for two identical metallic meander structures serving as an RTD (resistance temperature device): here, one element was activated by a catalyst and the other was only covered by a polymer as a passivation layer. Upon exposure to high-temperature gaseous H_2_O_2_ (of different concentrations), the catalyst (in this case, MnO_2_) reacted with the H_2_O_2_ and decomposed it into water and oxygen, i.e., fully environmentally friendly end products (Figure 2b).

The heat produced from this reaction was measured by the activated RTD on the sensor setup, using Equation (1):

2 H_2_O_2_ —> 2 H_2_O + O_2_, ΔH = −105.3 kJ/mol (1)

The difference between the temperatures measured by the active and passive elements represented the sensor signal, which corresponded to the H_2_O_2_ concentration. For the calorimetric gas sensor, which was mounted on the multi-sensing platform, the signals were read out with a sampling rate of 1 Hz by means of a National Instruments measurement card (NI 9219) together with an adapted software (LabVIEW 2017). The assessment of the temperature and concentration of the H_2_O_2_ gas using the calorimetric gas sensor was a step toward the validation of the optimum sterilization conditions as it had a correlation with the logarithmic kill rate of the spores (which is discussed in the results section).

For the detection of the relative humidity in the sterilization chamber containing the gaseous H_2_O_2_, a SHT31-D sensor (Sensirion AG) was applied to the multi-sensing platform (Figure 1, top left). The sensor was able to withstand short-term exposures to temperatures above 120 °C and monitored the relative humidity in the chamber during the whole sterilization process (i.e., under the various scenarios mentioned in Table 1). A micro-controller board (Arduino Duemilanove, Arduino IDE 1.8.15) was employed to read out the sensor values. By taking advantage of the humidity assessment, the change of relative humidity in the chamber was also monitored and its relationship to the sterilization validity was determined as a correlation toward the logarithmic kill rate of the spores.

The H_2_O_2_ gas flow detection was assessed by means of a specifically designed sensor, which fulfilled the conditions for the gas flow measurements (Figure 1, bottom right). An arrangement of four Pt100 temperature elements (Heraeus Nexensos SMD, Conrad Electronics), which were soldered on the PCB platform, was used_._

For the calibration of this arrangement, the PCB was placed in a 3D-printed PCB holder, which was made in-house, and fixed in such a way that any slipping and moving of the plate was avoided. This housing could also withstand the harsh conditions inside the sterilization chamber. A flexible test tube was used to allow the gas (at room temperature) to flow in different directions through the device in such a way that the changes in flow direction could occur and be detected with the Pt100 temperature sensors on the PCB. Without additional gas flow, the Pt100 elements initially registered the same temperature. However, as the H_2_O_2_ gas flow was applied, the distributed temperature profile changed, which led to a change in the resistance of the respective elements or among each other.

For the sterilization experiments using gaseous H_2_O_2_, the PCB was placed below the H_2_O_2_ outlet nozzle in the experimental test rig, according to the description in [35]. A data logger (Expert Logger 100, Delphin Technology AG), together with the adapted LabVIEW software, was used to process the flow characterization data from these experiments. The flow direction could, therefore, be determined by the observation of the temperature changes. The evaluation of the gas flow in the sterilization chamber was important. The influence of this factor on sterilization is also discussed in the results section as a correlation with the logarithmic kill rate of the spores.

In parallel to the characterization of the multi-sensing platform under various conditions, the spore viability during the sterilization process was also evaluated using count reduction tests as a reference method. A series of experiments was designed using resistant spores of *B. atrophaeus* DSM 675 with a starting germ count (N_0_) of 10^6^ colony-forming units (CFU)/mL under varying conditions. The spores, which were immobilized on a glass substrate (similar to spore immobilization on the biosensor as discussed in [19]), were placed in the sterilization chamber and exposed to the various concentrations of gaseous H_2_O_2_ (0, 2.2, 4.1, 5.7, 7.1 and 7.7% *v/v*) for very short time intervals (0.2, 0.4 or 0.6 s). Two different gas flow rates (8 and 12 m^3^/h) and three different gas temperatures (210, 240 and 270 °C) were selected for the evaporated hydrogen peroxide used in these microbiological experiments (also see Table 1). The values corresponded to the dose rates that are typically applied in industrial processes. It has to be noted that at a H_2_O_2_ gas flow of 12 m^3^/h, a maximum H_2_O_2_ concentration of 6.6% *v/v* could be used due to the limitations of this experimental setup.

In the count reduction test, the ratio between the starting (N_0_) and final germ count (N) of the spores is referred to as the logarithmic kill rate (LKR = log(N_0_/N)) in relation to the varying parameters. Large LKR values indicate that more spores have been killed and a more reliable sterilization has been performed (i.e., an LKR of 6 implies that from an initial spore count of 10^6^, the final germ count has been reduced to one).

The count reduction test series was performed for the various scenarios defined in Table 1 and the spore kill rates for the critical process parameters were evaluated. Finally, the correlation between the spore kill rates and the different sterilization conditions was assessed to acquire a valid statement for successful sterilization (which is discussed in the results section).

## 3. Results and Discussion

### 3.1. Calorimetric Gas Sensor

**Figure 3:**
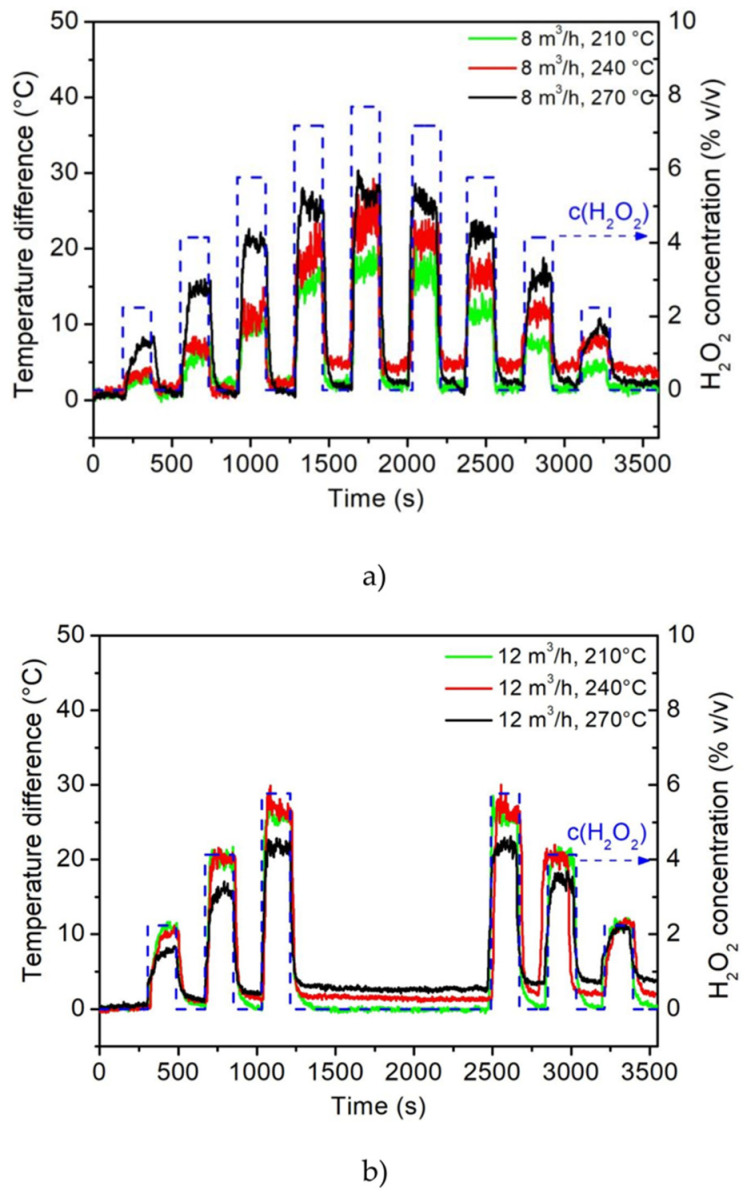
Assessing the calorimetric gas sensor signals and, consequently, the H_2_O_2_ concentrations (0, 2.2, 4.1, 5.7, 7.1/6.6 and 7.7% *v/v*) under varying gas flow rates (8 m^3^/h (**a**) and 12 m^3^/h (**b**)) at temperatures of 210, 240 and 270 °C, respectively.

The calorimetric gas sensor in the multi-sensing platform was used to determine the temperature and concentration of the H_2_O_2_ at the gas flow velocities of 8 m^3^/h (Figure 3a) and 12 m^3^/h (Figure 3b) when applying the different gas temperatures of 210, 240 and 270 °C. At the same time, the H_2_O_2_ concentration varied between 0 and 7.7% *v/v* (the blue dashed line in Figure 3a,b at the right-hand *y*-axis). The sensor was exposed to each concentration of gaseous H_2_O_2_ for 180 s and, after each exposure, to hot air for 180 s (at corresponding temperatures of 210, 240 and 270 °C) to re-equilibrate.

For a gas flow of 8 m^3^/h, the increase in the temperature difference (i.e., the sensor signal) when the initial gas temperature rose from 210 to 270 °C (Figure 3a) can be recognized. Here, the active element of the sensor reacted more with the gaseous H_2_O_2_ at higher temperatures and the difference between the active and passive elements became more pronounced. This can be explained by the sensor’s sensitivity and its correlation with the gas temperature.

In previous research, the relationship between the gas temperature and the sensitivity of the gas sensor has been studied [36]. At lower gas temperatures, the sensitivity decreases according to the Arrhenius equation, as described in that study. This effect can also be clearly observed in the temperature measurements here: the sensitivity of the calorimetric gas sensor at 210, 240 and 270 °C was evaluated as 1.8, 1.9 and 3.5 °C/% *v/v*, respectively. At a gas flow velocity of 12 m^3^/h, the sensor signal was higher at 210 and 240 °C in comparison to the same temperatures at a gas flow of 8 m^3^/h (Figure 3b). This effect can be explained by the heat dissipation on the sensor surface, which correlated with the gas flow rate [36].

At a gas temperature of 270 °C, the sensor signal decreased (Figure 3b) in comparison to the signals at gas temperatures of 210 and 240 °C. This effect could be explained by the fact that, with the high temperature at a gas flow of 12 m^3^/h, the passive element heated up as well as the active element; therefore, the temperature difference did not increase in comparison to the same cases at 210 and 240 °C. This was also noticeable in the raw data. In addition, the heat transmission between the two temperature elements was higher, which led to the passive temperature element heating up. 

The application of the calorimetric gas sensor in the multi-sensing platform enabled the online detection of the temperature and concentration of the gaseous H_2_O_2_. The response time (t_90_) of the temperature elements was evaluated to be <30 s, which allowed for online reporting. The continuous control of the temperature and concentration of the gas inside the aseptic chamber is a key step toward maintaining the best conditions to guarantee the highest spore kill rate in the sterilization process.

### 3.2. Humidity Detection

**Figure 4:**
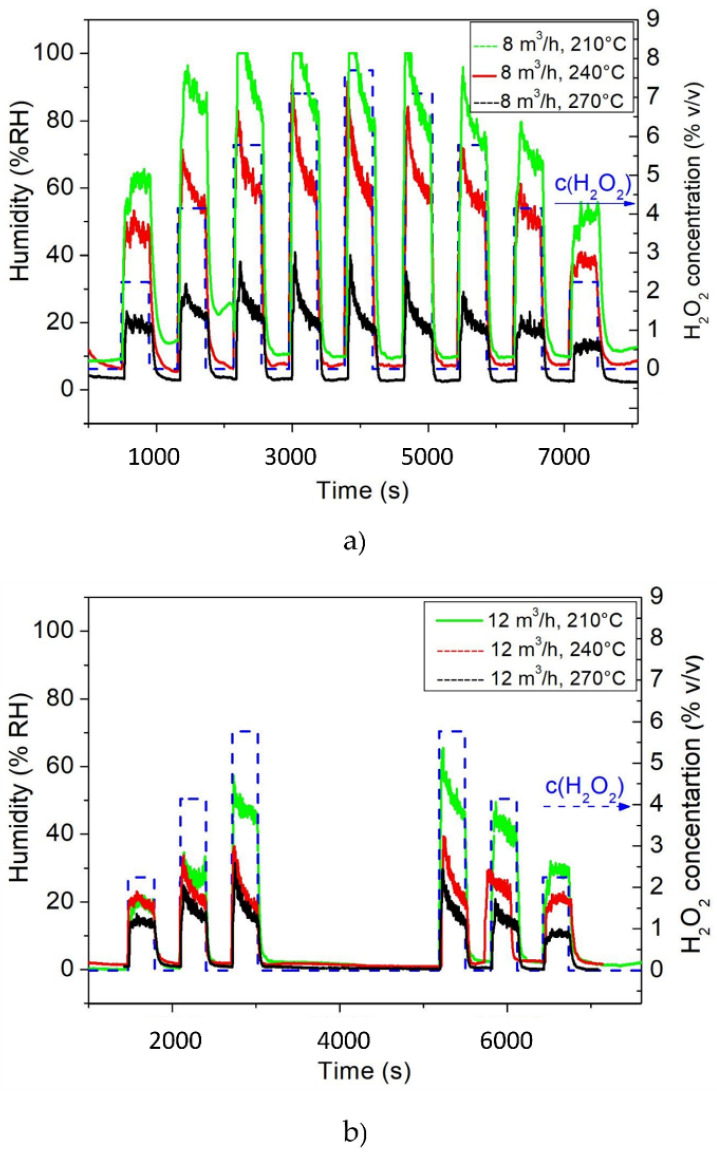
Assessing the relative humidity changes under the varying gas flow rates of 8 m^3^/h (**a**) and 12 m^3^/h (**b**) at temperatures of 210, 240 and 270 °C with H_2_O_2_ concentrations of between 0 and 7.7% *v/v*.

The relative humidity inside the aseptic chamber was monitored during the sterilization process using a SHT31-D humidity sensor. The results from the humidity measurements under the different conditions are presented in Figure 4.

Here, several concentrations of H_2_O_2_ were chosen (see scenarios in Table 1). The applied H_2_O_2_ was 35% *w/w*, meaning that the H_2_O_2_ was diluted by water to 35%. Yet, to adjust the water content of the volume, more information was required. The evaporated H_2_O_2_ is carried by air, hence, the output flow was a mixture of H_2_O_2_, water and air. For instance, when an output H_2_O_2_ concentration of 2.2% *v/v* was set, 2.2% of one volume unit of the gas (which flowed with the velocity of 8 m^3^/h) was pure H_2_O_2_. The rest was made of air and water. On the one hand, H_2_O_2_ of 35% *w/w* was used, so the remaining weight content was water. On the other hand, air was the carrier of H_2_O_2_ and as such, the remaining volume content (in 1 L) was air. This information allowed us to consider the different concentrations of H_2_O_2_, keeping in mind the volume of water in the gas flow. Table 2 shows some examples of the relative water content of different H_2_O_2_ concentrations in the gas stream when using H_2_O_2_ of 35% *w/w*. The higher the H_2_O_2_ concentration, the higher the water content of the gas mixture became, as more water–H_2_O_2_ mixture was used to obtain the set concentration. Obviously, concentrations other than 35% *w/w* H_2_O_2_ were also used, so those values differed accordingly.

It is visible in Figure 4a,b that the increase in H_2_O_2_ concentration led to an increase in humidity up to the stage of saturation. At the gas flow velocity of 8 m^3^/h, the overall rate of humidity decreased with the increase in the gas temperature (Figure 4a). A similar situation occurred for a gas flow of 12 m^3^/h (Figure 4b). It can be observed that, with a H_2_O_2_ concentration of 5.7% *v/v* at a gas temperature of 210 °C and a gas flow of 12 m^3^/h, the humidity was lower (~55% RH) in comparison to the same gas temperature at 8 m^3^/h (~99% RH). Additionally, for the gas temperatures of 240 and 270 °C, the humidity decreased further to 80% RH and 30% RH for 8 m^3^/h and 35% RH and 30% RH for 12 m^3^/h, respectively. The humidity sensor was implemented in the multi-sensing platform to monitor the moisture content inside the aseptic chamber, since humidity also has an influence on the quality of sterilization [32,37]. According to the above-mentioned study, a higher content of water inside the aseptic chamber could lead to the condensation of water droplets, which could result in a lower logarithmic kill rate of the spores [32]. In this regime, the online monitoring of the relative humidity inside the sterilization chamber could help to maintain the relative humidity at the most efficient level for achieving the highest spore kill rate throughout the sterilization.

### 3.3. Gas Flow Direction

**Figure 5:**
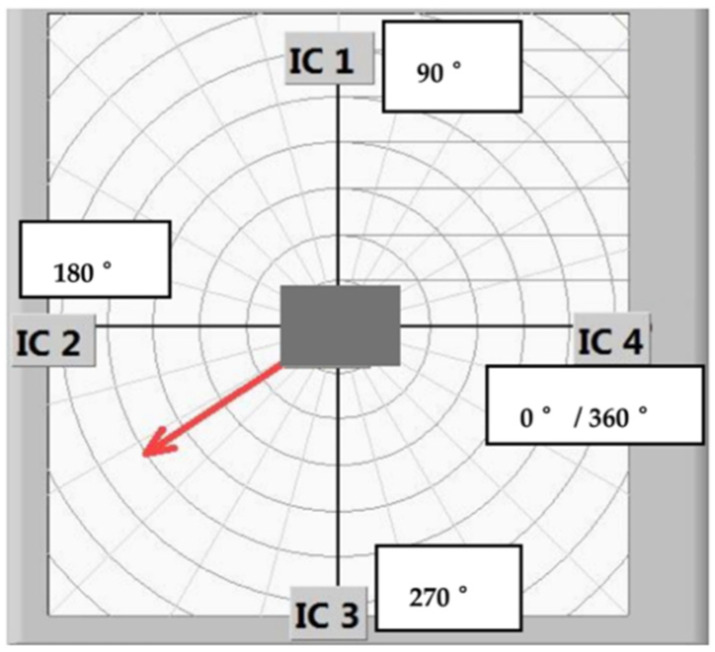
The detection of the H_2_O_2_ flow direction by means of the square arrangement of four Pt100 elements, IC 1 to IC 4.

The determination of the gas flow direction during the sterilization experiments using gaseous H_2_O_2_ was investigated with a configuration of four Pt100 elements on the PCB; namely, IC 1–4, Figure 5. The arrangement of these Pt100 temperature sensors enabled the measurement of a rise or fall in temperature when the gas flow increased or decreased. The flow direction was calculated from the temperature profiles of the Pt elements. For instance, as the H_2_O_2_ nozzle directed a flow between IC 2 and IC 3 (see the red arrow in Figure 5), the temperature profile between IC 1 and IC 4 would be different from that being streamed (between IC 2 and IC 3). As a result, the flow direction could be obtained from the discrepancy between the different temperature profile configurations.

**Figure 6:**
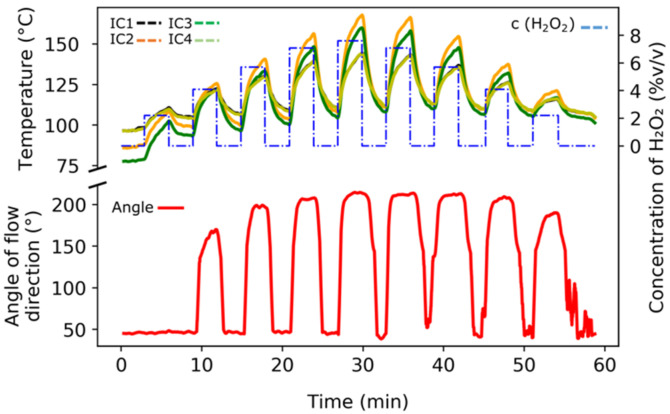
Assessing the change of gas flow direction under a H_2_O_2_ flow rate of 8 m^3^/h at 270 °C.

In the sterilization experiments using H_2_O_2_, the PCB was mounted under the gaseous H_2_O_2_ nozzle in the aseptic chamber. Figure 6 shows the evaluation of flow direction during the sterilization process using gaseous H_2_O_2_. In the upper part of the diagram, the temperature values of the four temperature sensors (IC 1–IC 4 with colors shown in the legend) with regards to their respective H_2_O_2_ dosage (the blue dashed curve) at a gas flow rate of 8 m^3^/h are plotted for a selected gas temperature of 270 °C. IC 1 and IC 4 were placed in the opposite direction of the gas flow (which was toward IC 2 and IC 3). As a consequence, the signals in the diagram are very similar and are difficult to distinguish from each other.

It is noticeable that the temperature curves are slightly different, which illustrates the direction dependence of the flow. A similar behavior was found for a flow rate of 12 m^3^/h (data not shown). When the flow was directed from IC 1 and IC 4 toward IC 2 and IC 3, the former elements were colder (as can be seen in Figure 6) than the latter elements. This indicates that the direction of flow was between IC 2 and IC 3. In addition, the angles of the flow direction are shown (in the lower part of Figure 6) by the red measurement curve that was calculated and recorded in the LabVIEW software during the measurement.

It should be noted that an angle of 200° (also shown schematically in Figure 6) did not mean a large deflection of flow from the outlet (positioned at 0°), but the schematic view indicates very precisely (using the detected temperature differences of the Pt100 elements) that the gas flow was slightly away from a straight line at 180° in the polar axis. However, it is also important to appreciate that the flow direction did not fluctuate during the aseptic process in the test rig. The change of the flow direction in the aseptic processes induced a change in the gas temperature, which consequently affected the outcome of spore sterilization. Therefore, in this regime, it was helpful to also monitor the gas flow direction in the aseptic chamber online to ensure a valid kill rate of the spores.

### 3.4. Spore Sterilization/Viability

**Figure 7:**
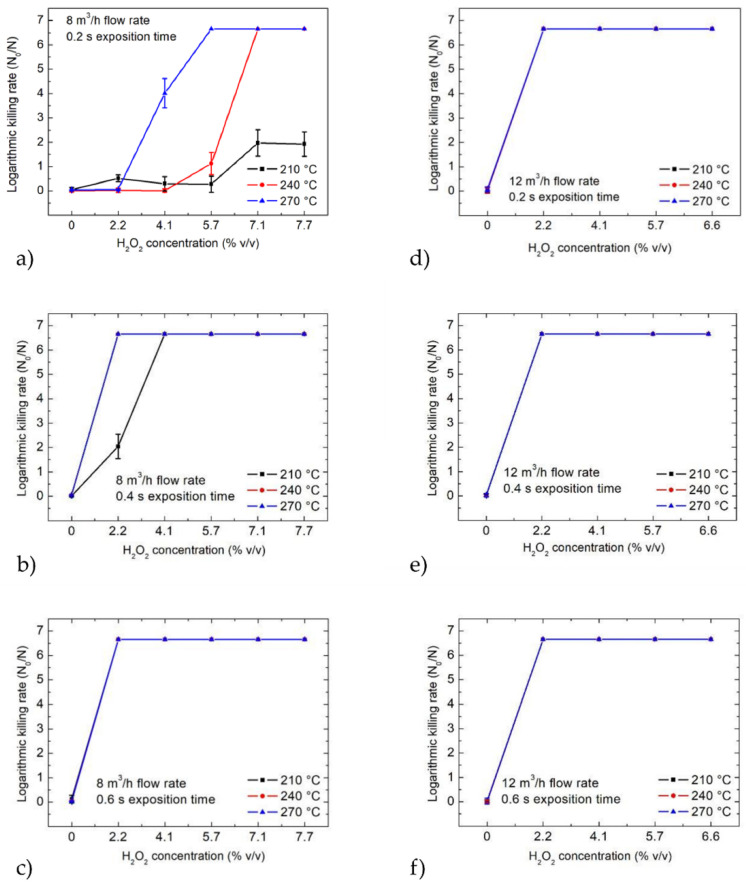
Spore viability validated by count reduction testing. The spores were sterilized in different scenarios. The H_2_O_2_ sterilization of *B. atrophaeus* DSM 675 with a gas flow rate of 8 m^3^/h (**a**–**c**) and with a gas flow rate of 12 m^3^/h (**d**–**f**), with varying exposure times of 0.2, 0.4 and 0.6 s at gas temperatures of 210, 240 and 270 °C with H_2_O_2_ concentrations of between 0 and 7.7 or 6.6% *v/v*.

For the count reduction tests that were used as a microbiological reference method, spores of *B. atrophaeus* DSM 675 were immobilized onto glass substrates with a spore count of 10^6^ per chip. The spores were exposed to various concentrations of hydrogen peroxide under the same conditions and scenarios as the multi-sensing platform (see Table 1, Figure 7).

The experiments were carried out at three gas temperatures (210 °C, 240 °C and 270 °C), while H_2_O_2_ concentrations were applied from 0 to 7.7% *v/v* for the 8 m^3^/h gas flow and up to 6.6% *v/v* for the 12 m^3^/h gas flow. The logarithmic kill rates for the count reduction tests were calculated after the sterilization.

Figure 7a–f presents the results of the sterilization process (i.e., the logarithmic kill rate of the spores) at the gas flow rates of 8 and 12 m^3^/h and with the exposure times of 0.2, 0.4 and 0.6 s. Figure 7a–c shows that at a temperature of 210 °C, the spores could survive for an exposure time of 0.2 s, even at the highest applied H_2_O_2_ concentration of 7.7%. A reliable sterilization and complete killing of the spores in 0.2 s could be achieved at gas temperatures of 240 °C and 270 °C with the corresponding minimum required H_2_O_2_ concentrations of 7.1 and 5.7% *v/v*, respectively (Figure 7a). In the case of the 8 m^3^/h gas flow, longer exposure times of 0.4 and 0.6 s presented better results for the sterilization of the spores (Figure 7b,c).

In Figure 7d–f, the results from a gas flow rate of 12 m^3^/h are presented. Figure 7d indicates that even the lowest applied H_2_O_2_ concentration of 2.2% *v/v* and the shortest exposure time of 0.2 s were sufficient to sterilize all spores at all three gas temperatures (210, 240 and 270 °C). Similar results were achieved for the exposure times of 0.4 and 0.6 s (Figure 7e,f).

The microbiological experiments served as a reference method for the validation of the sterilization. The results of these measurements were compared to the outcomes derived from the multi-sensing platform to correlate the physical and microbiological data and conclude the successful sterilization conditions. The correlation of these parameters is discussed in the next section to elaborate on the qualitative model used to predict the sterilization status.

### 3.5. Correlation between Process Parameters

**Figure 8:**
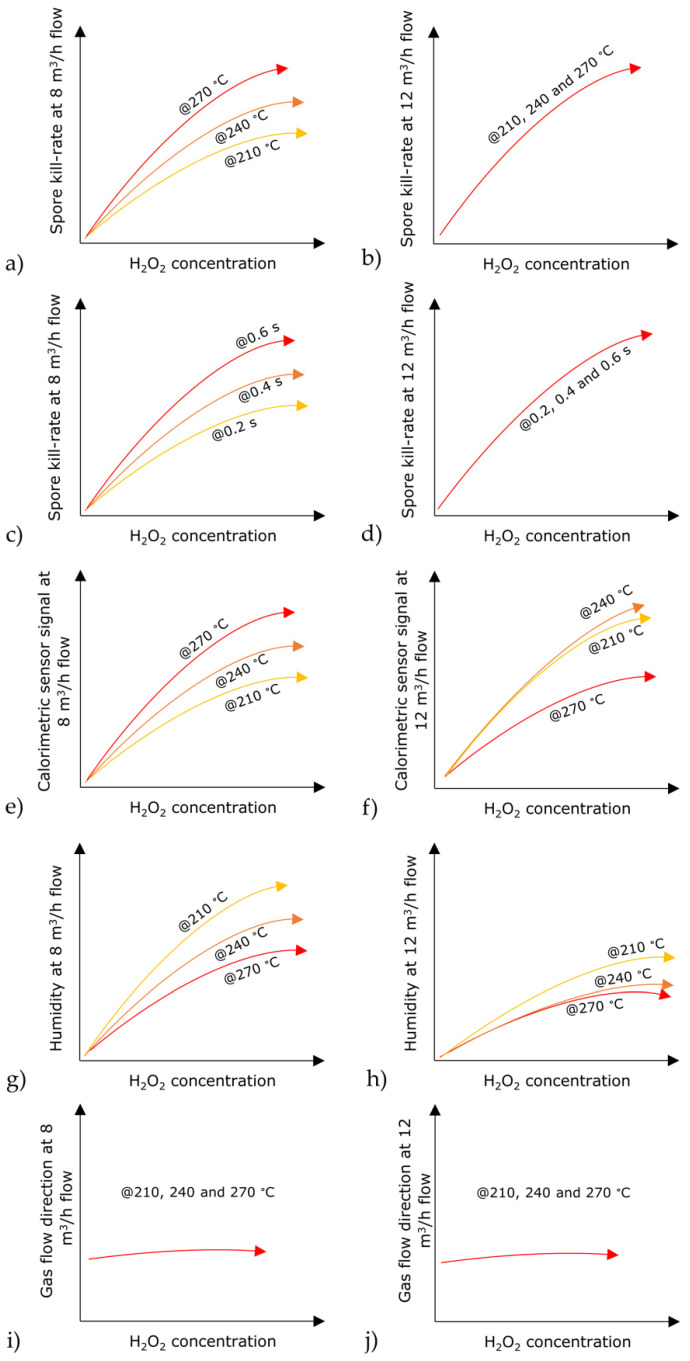
A schematic presentation of the correlations between the critical parameters during the sterilization process, detected by the multi-sensing platform, and the spore kill rates corresponding to the H_2_O_2_ concentration during the sterilization process. (**a**–**d**) The kill rate of *B. atrophaeus* DSM 675 spores; (**e**,**f**) the detection of temperature changes by the calorimetric gas sensor; (**g**,**h**) the relative humidity; and (**i**,**j**) the H_2_O_2_ gas flow direction under gas flow rates of 8 and 12 m^3^/h, respectively.

Various parameters of the sterilization process, such as H_2_O_2_ concentration and temperature, exposure time, humidity and gas flow direction, have distinct impacts on the optimization of the sterilization conditions [19,32,36]. In this study, these critical parameters were investigated simultaneously, in parallel with microbiological reference experiments. Specific relationships between the individual process parameters could be identified. These correlations enabled a (qualitative) predictive “modelling” between the parameters and various H_2_O_2_ concentrations that were used, which are schematically presented in Figure 8.

First, and most important, was the relationship between the spore kill rate and the H_2_O_2_ concentration, which is shown in Figure 8a,b for a H_2_O_2_ gas flow of 8 and 12 m^3^/h, respectively. It was found that with a H_2_O_2_ gas flow of 8 m^3^/h, higher gas temperatures (among 210, 240 and 270 °C) were more effective. While, at a H_2_O_2_ gas flow of 12 m^3^/h, the efficient killing of *B. atrophaeus* DSM 675 spores under H_2_O_2_ concentrations took place, regardless of the selected gas temperature (i.e., 210, 240 and 270 °C) (see also Figure 7d–f). In addition, it was also possible to define a correlation between the spore kill rate and the exposure time (0.2, 0.4 or 0.6 s) of the spores to the gaseous H_2_O_2_ (Figure 8c,d). For a gas flow of 8 m^3^/h, a longer exposure time led to a higher kill rate, whereas for a gas flow of 12 m^3^/h, the optimal kill rate was achieved within the shortest exposure time of 0.2 s. 

As a conclusion, when choosing the lower gas flow of 8 m^3^/h, higher gas temperatures and exposure times need to be applied. When utilizing the higher H_2_O_2_ gas flow of 12 m^3^/h, any of the indicated gas temperatures and exposure times can be applied and yet still obtain the efficient sterilization of the spores. One reason for this could be the less humid atmosphere inside the sterilization chamber, as humidity decreases the efficacy of sterilization when higher H_2_O_2_ concentrations are applied due to the condensation effect [32,37].

The calorimetric gas sensor detected an increase in the sensor signal due to the increase in H_2_O_2_ concentration and the exothermal decomposition of the H_2_O_2_ on the MnO_2_ sensor surface (Figure 8e,f). With the H_2_O_2_ gas flow of 8 m^3^/h, a higher signal from the calorimetric sensor was also recorded with the increasing gas temperatures (Figure 8e). With the increased H_2_O_2_ gas flow of 12 m^3^/h, the opposite sensor signal behavior was recorded at 270 °C (Figure 8f). As described previously, the passive temperature element also heated up due to the higher gas flow, which led to a reduced sensor signal. However, this decrease in sensor signal did not negatively influence the spore sterilization, as a successful kill rate was confirmed at 270 °C and with a 12 m^3^/h gas flow (indicated in Figure 7d–f and Figure 8b).

Figure 8g,h indicates a humidity increase due to the increase in H_2_O_2_ concentration since the water content of the gaseous H_2_O_2_ increased as well, as explained previously (see also Table 2). However, at a gas flow of 8 m^3^/h, the humidity decreased with the increasing gas temperature (210, 240 and 270 °C). A similar situation also occurred for a gas flow of 12 m^3^/h. The drier conditions in the sterilization chamber were beneficial for achieving a valid sterilization, as the humidity in higher H_2_O_2_ concentrations could result in condensation forming as water droplets and affecting the efficacy of the sterilization of the spores, as discussed above [32].

To conclude, lower humidity, which results from a higher temperature and gas flow, is beneficial for a successful sterilization. Apart from the benefit of the lower humidity in the aseptic process, it is also helpful in removing the H_2_O_2_ residue from the surfaces after the sterilization process has ended [5].

As expected, the gas flow direction remained unchanged when increasing the H_2_O_2_ concentration (Figure 8i,j). A constant flow direction was maintained as soon as a H_2_O_2_ concentration of above 4.1% *v/v* was applied, regardless of the gas temperature or gas flow rate used. The uniformity of the gas flow direction is important for spore sterilization, as a change in the direction could lead to a change in the gas temperature, H_2_O_2_ concentration or humidity; as a result, the potency of the aseptic process would be compromised.

Generally, by using the higher gas flow rate of 12 m^3^/h, the gas flow direction was more stable, which had a direct influence on the more effective sterilization of the spores. For a gas flow of 8 m^3^/h, it was beneficial to apply a longer exposure time; in that way, effective conditions for achieving the highest kill rate of spores can be obtained. A qualitative assessment of these correlations is summarized in Table 3.

All of the above-mentioned sterilization process parameters were evaluated simultaneously using the presented multi-sensing platform. The sensor results were correlated with the data from the time-consuming and laborious count reduction tests (using *B. atrophaeus* DSM 675 resistant spores), which showed a high conformity. Once the results from the microbiological tests were established, the necessary settings could be applied to the sterilization chamber, which could then be continuously monitored using the multi-sensing platform without any further repetition of the microbiological tests. For example, with a constant flow of gaseous H_2_O_2_, the suitable corresponding temperature and exposure time could be set and controlled online. The flow direction could be constantly evaluated and the chamber humidity could be monitored in order to achieve all necessary conditions and maintain them on-site for a successful sterilization process. Subsequently, this novel multi-sensing platform is applicable in the sterilization process to monitor and control the critical process both online and on-site and, at the same time, to avoid additional time-consuming and costly microbiological experiments.

## 4. Conclusions

A novel platform for the online monitoring of food package sterilization conditions was introduced to validate various parameters. This enabled us to control and record variations of the most effective parameters to obtain the highest kill rate of *B. atrophaeus* spores. One single board integrating multiple sensor setups was fabricated and mounted to a test rig for the sterilization process, including the necessary measurement electronics as well as software tools.

Calorimetric gas sensors were used because of their reliability in detecting the gas temperature and H_2_O_2_ concentration. The SHT31-D humidity sensor assessed the relative humidity inside the sterilization chamber and the Pt100 element arrangement was mounted on the board as well to monitor the gas flow direction. Multiple parameters within the sterilization process were successfully detected and measured using the fabricated multi-sensing platform. In addition, one round of microbiological experiments was performed under the same sterilization conditions, utilizing the resistant spores of *B. atrophaeus* DSM 675, in order to confirm the validity of the sterilization process. The data acquisition of this unique setup was provided by dedicated electronics to read out and evaluate the data from the various sensors on the board.

This cooperation of sensor designs on one single multi-sensing platform allowed the more specific detection of manifold input parameters in aseptic filling machines compared to the traditional microbiological methods. The new setup is, therefore, input-related and it avoids large time delays, which are known for output-related methods (e.g., count reduction testing). The new multi-sensing platform provides several benefits, which could solve the present challenges in industry: it offers online and on-site monitoring, the problem with sample preparation could be solved and there would be no need for cell culture testing. Consequently, the food package industry would save valuable time and resources compared to the traditional microbiological tests. In that way, the high costs of the maintenance and laborious work of the traditional aseptic controls in the companies would be avoided because, as our results confirm, this platform can monitor aseptic conditions both online and on-site. Furthermore, the new setup enables a permanent logging of the aseptic parameters for the first time. This supports the industry’s need for continuous monitoring and verification and high-quality assurance.

## Figures and Tables

**Table 1:** A table of the varying conditions applied to the spore sterilization experiments.

c (H_2_O_2_) (% *v/v*)	Gas Flow Velocity (m^3^/h)	Gas Temperature (°C)	Time Intervals (s)
0	8	210	0.2, 0.4, 0.6
		240	0.2, 0.4, 0.6
		270	0.2, 0.4, 0.6
	12	210	0.2, 0.4, 0.6
		240	0.2, 0.4, 0.6
		270	0.2, 0.4, 0.6
2.2	8	210	0.2, 0.4, 0.6
		240	0.2, 0.4, 0.6
		270	0.2, 0.4, 0.6
	12	210	0.2, 0.4, 0.6
		240	0.2, 0.4, 0.6
		270	0.2, 0.4, 0.6
4.1	8	210	0.2, 0.4, 0.6
		240	0.2, 0.4, 0.6
		270	0.2, 0.4, 0.6
	12	210	0.2, 0.4, 0.6
		240	0.2, 0.4, 0.6
		270	0.2, 0.4, 0.6
5.7	8	210	0.2, 0.4, 0.6
		240	0.2, 0.4, 0.6
		270	0.2, 0.4, 0.6
	12	210	0.2, 0.4, 0.6
		240	0.2, 0.4, 0.6
		270	0.2, 0.4, 0.6
7.1/6.6	8	210	0.2, 0.4, 0.6
		240	0.2, 0.4, 0.6
		270	0.2, 0.4, 0.6
	12	210	0.2, 0.4, 0.6
		240	0.2, 0.4, 0.6
		270	0.2, 0.4, 0.6
7.7	8	210	0.2, 0.4, 0.6
		240	0.2, 0.4, 0.6
		270	0.2, 0.4, 0.6
	12	210	0.2, 0.4, 0.6
		240	0.2, 0.4, 0.6
		270	0.2, 0.4, 0.6

**Table 2:** An example of the relative water content in different gaseous H_2_O_2_ concentrations.

H_2_O_2_ (% *v/v*)	H_2_O (% *v/v*)	Air (% *v/v*)	Sum (%)
2.24	6.11	91.64	100
4.14	11.28	84.57	100
5.77	15.71	78.51	100
7.18	19.54	73.26	100
7.7	20.94	71.35	100

**Table 3:** A qualitative assessment of the correlations between the sterilization process parameters.

Sterilization of Spores	H_2_O_2_ Concentration (% *v/v*)	Gas Flow (m^3^/h)	Exposure Time (s)	Gas Temperature (°C)	Chamber Humidity (%)	Flow Direction
Successful	2.2–6.6	12	≥0.2	210, 240, 270	≤55	Constant
Successful	4.1–7.7	8	≥0.4	210, 240, 270	≤90	Constant

## Data Availability

The data presented in this study are available on request from the corresponding author.
